# NOTCH ligands JAG1 and JAG2 as critical pro-survival factors in childhood medulloblastoma

**DOI:** 10.1186/2051-5960-2-39

**Published:** 2014-04-07

**Authors:** Giulio Fiaschetti, Christina Schroeder, Deborah Castelletti, Alexandre Arcaro, Frank Westermann, Martin Baumgartner, Tarek Shalaby, Michael A Grotzer

**Affiliations:** 1 Department of Oncology, University Children’s Hospital of Zurich, Zurich, Switzerland; 2 Department of Tumor Genetics, German Cancer Research Center (DKFZ), Heidelberg, Germany; 3 Department of Clinical Research, University of Bern, Bern, Switzerland

**Keywords:** Medulloblastoma, NOTCH, JAG1, JAG2, Pediatric cancer

## Abstract

Medulloblastoma (MB), the most common pediatric malignant brain cancer, typically arises as pathological result of deregulated developmental pathways, including the NOTCH signaling cascade. Unlike the evidence supporting a role for NOTCH receptors in MB development, the pathological functions of NOTCH ligands remain largely unexplored. By examining the expression in large cohorts of MB primary tumors, and in established *in vitro* MB models, this research study demonstrates that MB cells bear abnormal levels of distinct NOTCH ligands. We explored the potential association between NOTCH ligands and the clinical outcome of MB patients, and investigated the rational of inhibiting NOTCH signaling by targeting specific ligands to ultimately provide therapeutic benefits in MB. The research revealed a significant over-expression of ligand JAG1 in the vast majority of MBs, and proved that JAG1 mediates pro-proliferative signals via activation of NOTCH2 receptor and induction of HES1 expression, thus representing an attractive therapeutic target. Furthermore, we could identify a clinically relevant association between ligand JAG2 and the oncogene MYC, specific for MYC-driven Group 3 MB cases. We describe for the first time a mechanistic link between the oncogene MYC and NOTCH pathway in MB, by identifying JAG2 as MYC target, and by showing that MB cells acquire induced expression of JAG2 through MYC-induced transcriptional activation. Finally, the positive correlation of MYC and JAG2 also with aggressive anaplastic tumors and highly metastatic MB stages suggested that high JAG2 expression may be useful as additional marker to identify aggressive MBs.

## Introduction

Medulloblastoma (MB) is the most common pediatric malignant brain cancer, accounting for approximately 20% of primary central nervous system neoplasms in this age group [1]. Because of the significant rate of mortality and treatment-related morbidity, further understanding of the molecular biology of MB is needed to improve current treatment regimens and discover novel and more effective molecular-targeted therapies. Four distinct MB subgroups have been identified based on common molecular alterations: WNT tumors are characterized by activated Wingless pathway and carry a favorable prognosis under current treatment regimens; SHH tumors, which possess active Sonic Hedgehog signaling, and Group 4 tumors, molecularly less well characterized, have an intermediate prognosis; Group 3 tumors are characterized by high levels of the oncogene MYC and associated with poor prognosis [2]. A molecular-based classification of MB is a crucial step towards optimized treatment schemes aiming at improving risk-benefit therapeutic profiles. One further layer of complexity is the identification of key biological alterations to be selectively targeted by tailored therapies.

MBs are heterogeneous cerebellar tumors, which commonly arise as the pathological result of deregulated developmental pathways, including the NOTCH cascade [3,4]. NOTCH signaling is required for the physiological development of the cerebellum during embryogenesis, controlling cell differentiation, proliferation, and apoptosis [5]. NOTCH cascade involves functionally non-redundant genes that appear to exert unique and specific functions [6]. Activation of the canonical NOTCH cascade requires the interaction of ligands (JAG1, JAG2, DLL1, DLL3, and DLL4) with receptors (NOTCH 1-4) [7]. Ligand binding triggers the proteolytic cleavage of NOTCH receptors, which is mediated by distinct enzymes, including γ-secretase. Once released into the cytoplasm, the NOTCH intracellular domain (NICD) translocates into the nucleus and activates a series of transcriptional regulatory events with context-dependent phenotypic consequences [7]. The spatial and temporal expression of receptors and ligands results in diverse heterogeneous cellular responses that can be cell- and tissue-specific, due to cross-talk with other pathways and the cellular microenvironment [8,9]. A growing body of evidence suggests that the ligands also have an intrinsic signaling activity, independent of canonical NOTCH, which may account for the pleiotropic effects of the NOTCH signaling [10].

Deregulation of NOTCH receptors and ligands has been described in a wide variety of human tumors, including pediatric malignancies, such as leukemia, glioblastoma, and neuroblastoma [11-15]. Given the important role of NOTCH signaling in both normal and pathologic cerebellum development, it is not surprising that defects in this pathway are also associated with MB development. In particular, oncogenic properties of NOTCH2 receptor have been associated with MB tumor proliferation, and high expression of the best-characterized NOTCH target gene, HES1, has been associated with poor clinical outcome [16,17]. However, to date the potential pathological functions of NOTCH ligands in MB remain largely unexplored. We hypothesized that the abnormal expression of NOTCH ligands in MB cells could trigger an alteration of the NOTCH cascade. Therefore, we investigated NOTCH ligands expression in MB primary samples and in *in vitro* MB models, we examined the potential association between NOTCH ligands and the clinical outcome of MB patients, and explored the rational of inhibiting NOTCH signaling by targeting specific ligands to ultimately provide therapeutic benefits in MB.

## Materials and methods

### Human MB primary samples and human-derived MB cell lines

The tumor material used in this study originates from archival MB samples from patients treated at the University Children’s Hospital of Zürich, Switzerland (n = 47, formalin-fixed paraffin-embedded MB samples). All tissue specimens used were obtained from the Swiss Pediatric Oncology Group (SPOG) Tumor Bank. Written informed consent was obtained from each patient by the hospital that provided the tissue samples. The use of SPOG Tumor Bank tissue samples for cancer research purposes was approved by the Ethical Review Board of Zurich (Ref. Nr. StV-18/02). MB cell lines were cultured as previously published [18,19] and maintained at 37°C in a humidified atmosphere with 5% CO2. DAOY human MB cells were purchased from the American Type Culture Collection (ATCC - Manassas, VA, USA). D341, D425, UW-228-2, and Med-1 human MB cells were the kind gift of Dr. Henry Friedman (Duke University, Durham). The stable clones DAOY V11 (empty vector transfected) and DAOY M2.1 (MYC vector transfected) were maintained in selective medium in the presence of 500 mg/ml G418 [20]. MB cells were grown as neuro-spheres in neurobasal medium added with B-27 Supplement (GIBCO - Life Technologies Grand Island, NY, USA), recombinant human EGF (20 ng/ml), and basic FGF (10 ng/ml) (R & D Systems Inc., Minneapolis, MN 55413 USA) in corning ultra-low binding 24-well plates (Sigma-Aldrich, St. Louis, MO, USA).

### Gene expression profiling studies of MB primary samples

MB expression profiles were generated on Affymetrix 133A [21]; Affymetrix 133plus 2.0 [22,23]; Affymetrix Human Gene 1.1 ST [24]; or Affymetrix exon 1.0 arrays [25,26]. The datasets used in this study were comparable regarding most patient characteristics [27]. The human normal cerebellum expression profile was generated with Affymetrix 133plus 2.0 [28]. Data are accessible through the open access platform R2 for visualization and analysis of the microarray data (http://r2.amc.nl).

### RNA analysis by qRT-PCR

Total RNA was extracted using the RNeasy Mini Kit (Qiagen, Basel, Switzerland) following the manufacturer’s instructions. After enzymatic digestion of DNA with RNase-free DNase (Qiagen), 0.5-1 μg of total RNA was used as the template for reverse transcription employing random hexamer primers and the High-Capacity cDNA Reverse Transcription Kit (Applied Biosystems - Life Technologies Grand Island, NY, USA). For the qRT-PCR reaction, Gene Expression Master Mix (Applied Biosystems) was used, and the protocol was optimized for the ABI7900HT reader (Applied Biosystems). Probe-primer solutions specific for the following genes (purchased from Applied Biosystems) were used: *MYC* (Hs00153408_m1), *JAG2* (Hs00171432_m1), *JAG1* (Hs01070032_m1), and *HES1* (Hs00172878_m1). Normal human adult cerebellum mRNA samples (Clontech-Takara Bio Europe, Saint-Germain-en-Laye, France) (R12340039-50, AMS Biotechnology Limited, 184 Park Drive, Milton Park, Abingdon OX14 4SE, UK.) and normal human fetal (40 weeks) cerebellum mRNA samples (R1244039-50, AMS Biotechnology) were used as a reference. The relative gene expression was calculated for each gene of interest using the ΔΔCT method, in which cycle threshold (CT) values were normalized to the housekeeping genes *succinate dehydrogenase complex subunit A* (*SDHA*) (Hs00188166_m1) and *18 s* (Hs99999901_s1).

### ChIP-on-chip analysis

Genomic DNA was extracted and precipitated with a MYC-specific antibody to enrich MYC-binding promoter sequences, which were hybridized to a promoter oligo-array as previously described [29]. The genomic positions for probes and their enrichment ratios are provided for MYC at the *JAG2* locus. The horizontal red line indicates the median enrichment ratio for MYC versus the input, as calculated from all probes for chromosome 14.

### Western blot analysis

Total protein extracts were obtained from 0.5-1.5 × 10^6^ cells lysed with RIPA buffer (50 mM Tris-Cl, pH 6.8, 100 mM NaCl, 1% Triton X-100, 0.1% SDS) supplemented with Complete Mini Protease Inhibitor Cocktail (Roche-Applied Sciences) and the phosphatase inhibitors β-glycerophosphate (20 mM) and Na3VO4 (200 μM). Proteins were resolved by sodium dodecyl sulfate polyacrylamide gel electrophoresis and blotted on PVDF membranes (GE Healthcare, Chalfont St Giles Buckinghamshire, UK) or Trans-Blot Turbo 0.2-μm nitrocellulose (Bio-Rad Laboratories, Inc., Hercules, CA 94547, USA). After binding of the primary antibodies, the signal was detected by chemiluminescence using SuperSignal West Femto Maximum Sensitivity Substrate (Pierce-ThermoScientific, Rockford, IL, U.S.A.). Antibody specific for Hes1 (H-140) (sc-25392) was purchased from Santa Cruz Biotechnology, Inc. (Santa Cruz Biotechnology, Inc. Santa Cruz, CA. U.S.A.); antibody specific for the Notch2 intracellular domain (Asp1733) (ab52302) was purchased from Abcam (Abcam plc, Cambridge, UK); antibodies specific for Jagged-2 (C23D2) (2210), JAG1 (28H8) (2620), cleaved Notch1 (Val1744), and MYC (9402) were obtained from Cell Signaling Technology (Cell Signaling Technology, Danvers, MA, U.S.A.). As a loading control, β-actin (Sigma-Aldrich, St. Louis, MO, U.S.A.) was detected by chemiluminescence using Pierce ECL Substrate (Pierce-ThermoScientific).

### siRNA transfection

Cells were transfected when they reached 70-80% confluence in 6-well plates using either SMARTpool small interfering RNA (siRNA) specific for *JAG2* (L-017187-00), *JAG1* (L-011060-00), and *MYC* (L-003282-00) or siCONTROL non-targeting siRNA pool (D-001810-10-05) as a control (Dharmacon, Thermo Fisher Scientific, Waltham, MA). Each pool of siRNA was used at the final concentration of 50 nM in combination with Dharmafect 4 as the transfection reagent (Dharmacon) according to the manufacturer’s instructions. After 24, 48, and 72 hours, the cells were harvested for both mRNA and protein extraction to assess gene expression by quantitative real-time PCR (qRT-PCR) and immunoblotting, respectively.

### Cell viability, proliferation, and apoptosis analysis

Viability of MB cells was evaluated using the CellTiter 96® AQueous One Solution Cell Proliferation Assay (Promega Corporation, Madison, WI, USA) and cell proliferation reagent WST-1 (05015944001) (Roche Diagnostics, Rotkreuz, Switzerland). Additionally, the number of viable cells was determined by trypan blue exclusion using a hemocytometer. Proliferation was quantified using the chemiluminescence-based Cell Proliferation ELISA BrdU kit (Roche Diagnostics). Activation of caspases 3 and 7 was detected using the Caspase-Glo 3/7 Assay (Promega Corporation). Histone-associated DNA fragments were quantified using Cell Death Detection ELISAPLUS assays (Roche Diagnostics). Data are expressed as the average values obtained from three independent experiments.

### Statistical analysis

All experiments were performed at least in triplicate. Data are represented as the mean ± s.d. For *in vitro* experiments, a Student’s t-test was used. P-values of <0.05 were considered significant. Pearson’s correlation test was used for gene correlation in patient samples. Student’s unpaired T and Mann-Whitney tests were applied for statistical analysis of normally and non-normally distributed samples, respectively [* < 0.05, ** < 0.01, *** < 0.001].

## Results

### NOTCH ligands are aberrantly expressed in medulloblastoma

**Figure 1:**
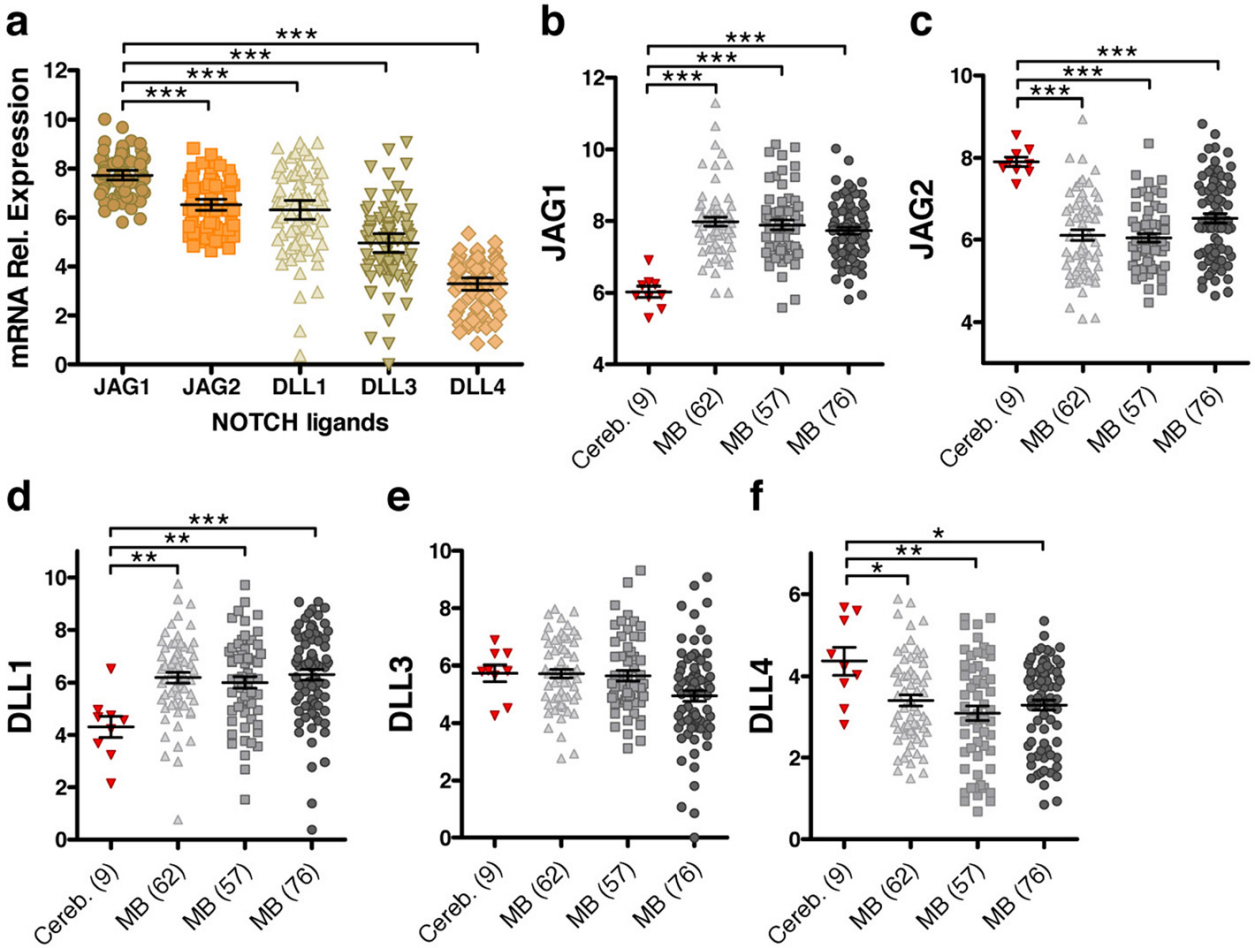
**Expression of NOTCH ligands in MB primary tumors. (a)** Relative mRNA expression of the indicated NOTCH ligands in a representative gene expression dataset of 62 human MB tumors [23]. Dot plots showing relative expression of *JAG1***(b)**, *JAG2***(c)**, *DLL1***(d)**, *DLL3***(e)**, and *DLL4***(f)** in three independent gene expression datasets of human MB tumors: 62 samples [23]; 57 samples [22]; 76 samples [26].

We first addressed whether MB tumors express predominantly one or more of the five known NOTCH ligands. By analyzing their expression in three distinct publicly available gene expression profiles of primary MBs (total of 195 cases), we showed that tumors samples express all five NOTCH ligands, with *JAG1* showing the highest expression levels compared to the other ligands (Figure 1a and Additional file 1: Figure S1a). The levels of *JAG2*, *DLL1*, and *DLL3* were comparable to each other, whereas that of *DLL4* was relatively lower. Our hypothesis that MB tumors bear aberrant levels of NOTCH ligands was verified when the expression levels in tumors were normalized to expression in normal cerebella (Figure 1b-f). The analysis revealed distinct significant aberrancies in the level of four ligands: *JAG1*, *JAG2*, *DLL1*, and *DLL4*. We observed an over-expression of JAG1 and DLL1 (Figure 1b and d, respectively) and a down-regulation of JAG2 and DLL4 (Figure 1c and f, respectively), whereas *DLL3* expression in MB tumor samples was comparable to that in cerebellum controls (Figure 1e). The most relevant change in expression was that of NOTCH ligand JAG1, which was significantly overexpressed in almost all MBs (189/195), compared to cerebellum. To a lesser extent, also the up-regulation of DLL1, as well as the reduced expression of *JAG2* and of *DLL4*, appeared to be common features of MB tumors. Notably, the variance of sample distribution was smaller for *JAG1* and *JAG2*, both in MB tumors and control samples, whereas the expression levels of *DLL1*, *DLL3*, and *DLL4* demonstrated greater variation (Figure 1a and Additional file 1: Figure S1a and b). Together these findings confirmed that MB tumors harbor abnormal levels of distinct NOTCH ligands, which likely results in a broad alteration of the fine-tuning regulation of the signaling pathway.

### JAG1 is a survival factor mediating activation of canonical NOTCH2 signals in medulloblastoma

**Figure 2:**
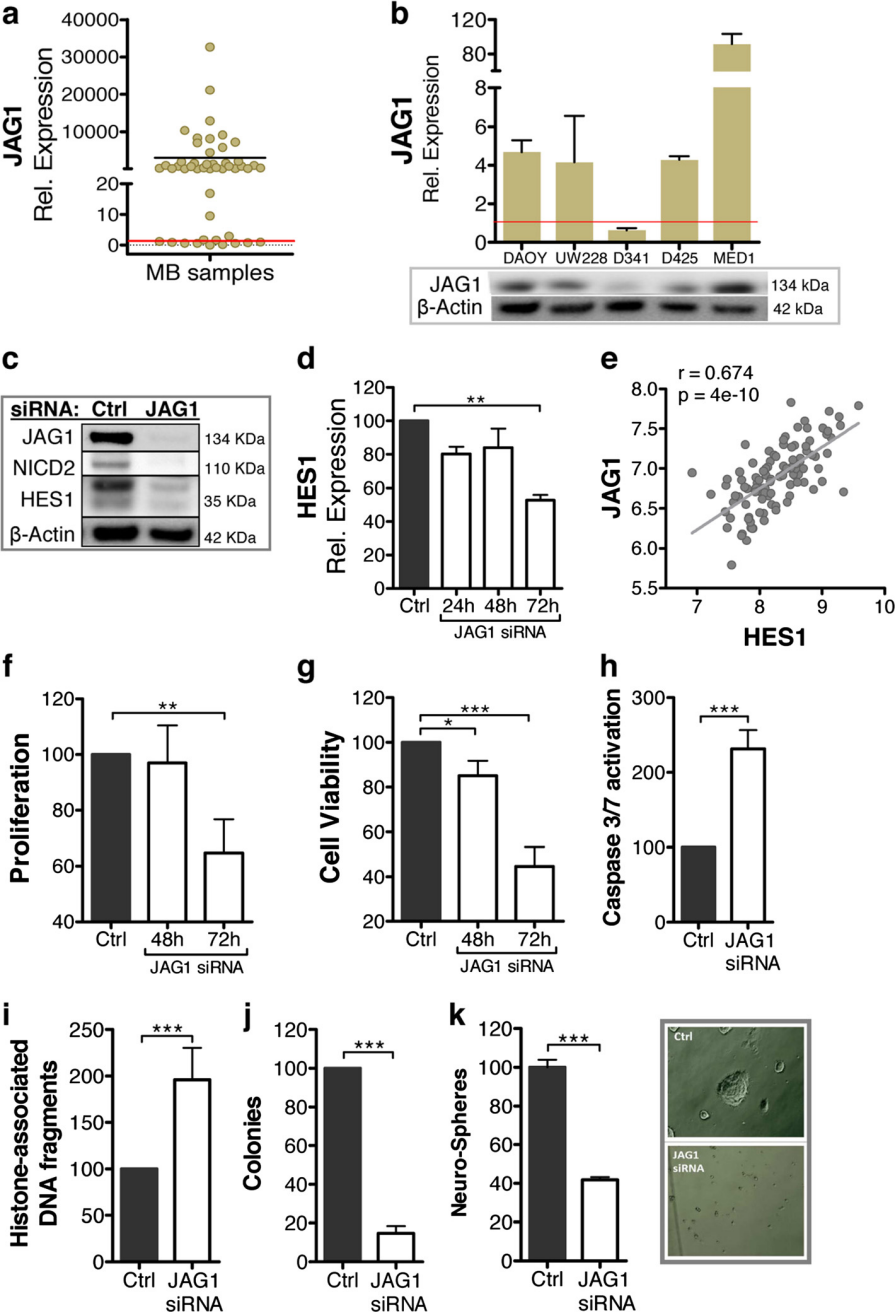
**JAG1 mediates pro**-**survival signaling through activation of canonical NOTCH2 signaling. (a)** Relative *JAG1* expression in 47 fresh frozen MB primary samples. Values represent the fold-change in *JAG1* mRNA expression compared to that in normal cerebellum samples (defined as 1). **(b)** JAG1 mRNA (upper panel) and protein (lower panel) expression in the indicated MB cell lines. mRNA values represent the fold-change in *JAG1* mRNA expression compared to that in normal cerebellum samples (defined as 1). **(c)** Protein expression of JAG1, NICD2, and HES1 in DAOY cells at 72 hours after treatment with JAG1 siRNA compared to control siRNA. **(d)***HES1* mRNA relative expression in DAOY cells upon JAG1 siRNA treatment at the indicated time-points. Values represent the percent decrease in *HES1* mRNA relative to the control. **(e)** Correlation between *JAG1* and *HES1* mRNA expression in a dataset of 103 MB tumors [25]. r: Pearson’s value; p: p values. Proliferation **(f)**, cell viability **(g)**, caspase 3/7 activation **(h)**, and histone-associated DNA fragments **(i)** of DAOY cells at 48 hours after JAG1 siRNA treatment or at the indicated time-points compared to control siRNA. Percent decrease in the number of colonies **(j)** and neuro-spheres **(k)** formed by DAOY cells 72 and 120 hours, respectively, following JAG1 siRNA treatment compared to control siRNA. (**k**, right panel) Representative image of DAOY-derived neuro-spheres upon treatment with JAG1 siRNA and control siRNA.

Because of the relevant broad over-expression of JAG1 in MB primary samples, we focused this section of the research on the analysis of the biological role of this specific NOTCH ligand. To validate the up-regulation observed in MB gene expression profiles, *JAG1* mRNA levels were quantified in an additional independent cohort of 47 MB primary samples collected at the Children’s Hospital of Zurich. Consistent with the previous findings, *JAG1* was overexpressed in 74% (35/47) of MB cases compared to normal cerebellum (Figure 2a). These findings were further verified by analyzing the protein and mRNA levels in a representative panel of human-derived MB cell lines. Four out of five cell lines overexpressed JAG1 compared to normal cerebellum (Figure 2b), confirming that MB cells bear aberrantly high levels of NOTCH ligand JAG1, also in established and characterized MB models. Notably, D341 MB cell line, which is genetically similar to tumors of the molecular Group 3 [30], showed a lower JAG1 expression level compared to the others (Figure 2b).

To determine whether the activation of the canonical NOTCH pathway in MB is mediated by JAG1, we quantified the levels of activated NOTCH receptors (NICD) and of the NOTCH target gene (HES1) in MB cells upon JAG1 depletion. siRNA-mediated silencing of JAG1 (Figure 2c and Additional file 2: Figure S2a) caused a drastic reduction in NICD2 and HES1 expression, at the protein and mRNA levels (Figure 2c and d, respectively), suggesting an important role for JAG1 in canonical NOTCH2 signaling activation. These results were further confirmed by comparative gene expression analysis of clinical MB samples, which showed a highly significant positive association between *JAG1* and *HES1* mRNA transcript levels (Figure 2e and Additional file 2: Figure S2b). Notably, NOTCH1 receptor did not appear to be affected by JAG1 depletion, since no relevant changes in NICD1 abundance could be detected (Additional file 2: Figure S2c). Therefore, these results depict JAG1 as important activator of pro-proliferative NOTCH2 cascade [17], and link a specific ligand to the level of HES1, whose high expression has been associated with poor prognosis [16,17]. Furthermore, without affecting NOTCH1, which has been described as anti-proliferative receptor in MB [17], JAG1 appears to specifically mediate pro-survival NOTCH2 signals in MB.

Indeed, JAG1 depletion negatively affected proliferation rate of MB cells (Figure 2f), and induced activation of the apoptotic machinery through enhanced caspase 3 and 7 activity, which led to an increase in apoptotic histone-associated DNA fragments and reduction of cell viability (Figure 2g, h, and i). Moreover, JAG1-depleted cells were unable to grow clonally (Figure 2j), and the capability of these cells to form neuro-spheres was reduced (Figure 2k), further supporting the notion that JAG1 has a key role in maintaining NOTCH-related pro-survival functions in MB cells.

Altogether, the relevant high expression level of JAG1 in the vast majority of tested MB samples, and the important role in promoting cell proliferation and survival, all pointed at JAG1 as a key player in canonical NOTCH activation and render this NOTCH ligand a potential target for novel selective strategies aimed at NOTCH inhibition in MB.

### Molecular subgroup-specific analysis reveals high levels of NOTCH ligand JAG2 in MYC-driven Group 3 tumors

**Figure 3:**
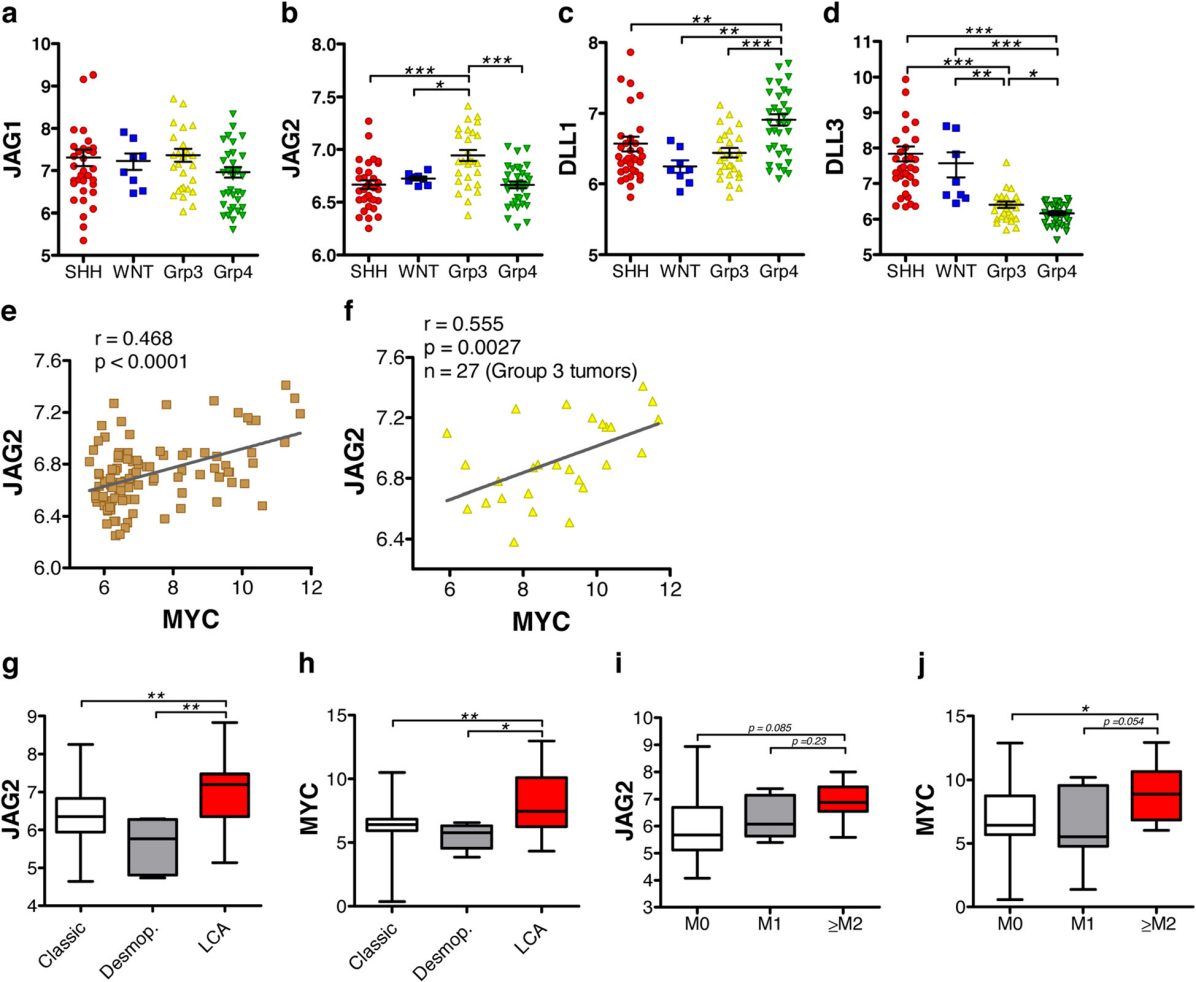
**Expression of NOTCH ligands across MB molecular subgroups and *MYC***/***JAG2 *expression correlation in MB primary tumors**, **in histological MB subtypes**, **and in distinct metastatic MB stages.** Dot plots showing relative expression (log2) of *JAG1***(a)**, *JAG2***(b)**, *DLL1***(c)**, and *DLL3***(d)**, across MB subgroups in a representative dataset of 103 human MB tumors [25]: SHH, n = 33; WNT, n = 8; Grp3 (Group 3), n = 27; Grp4 (Group 4), n = 35. **(e)** Correlation between *MYC* and *JAG2* mRNA expression (log2) in a representative dataset of 103 human MB tumors [25]. r: Pearson’s value; p: p values. **(f)** Correlation study of *JAG2* and *MYC* levels in Group 3 MB cases [25]. Box plots showing *JAG2***(g)** and *MYC***(h)** expression (log 2) according to the following MB histological variants [26]: classic (n = 51), desmoplastic (desmop.) (n = 6), and (LCA) large cells/anaplastic (n = 17); center line = median. Box plots showing *JAG2***(i)** and *MYC***(j)** expression (log 2) in MB tumors clustered according to metastatic stage [23]: M0, n = 42; M1, n = 7; ≥M2, n = 9; center line = median.

To date, the distribution and potential role of NOTCH ligands within the defined MB molecular sub-groups is unknown. We therefore examined NOTCH ligands expression levels across sub-groups in two distinct cohorts of MB primary samples with available annotation for molecular subgroups (n = 388) (Figure 3a-d and Additional file 3: Figure S3a-e). On one hand, the analysis confirmed that the overexpression of *JAG1* is a common feature across all four MB molecular subtypes (Figure 3a and Additional file 3: Figure S3a), thus highlighting the potential benefit of targeting JAG1 for the treatment of MB tumors of different origin. On the other hand, the study revealed distinct patterns of expression of other NOTCH ligands across subgroups. In particular, we noticed the presence of a subpopulation of Group 3 cases expressing high *JAG2* levels (Figure 3b and Additional file 3: Figure S3b), in contrast to the previous observation of a broad JAG2 under-expression in MB tumors (Figure 1c). Additionally, we could detect an up-regulation of NOTCH ligand *DLL1* in Group 4 cases (Figure 3c and Additional file 3: Figure S3c), and a down-regulation of *DLL3* in Groups 3 and 4 tumors (Figure 3d and Additional file 3: Figure S3d). No significant differences in *DLL4* expression were observed across the four MB subgroups (Additional file 3: Figure S3e).

Given the established oncogenic role of MYC in Group 3 tumors [23,31] (Additional file 3: Figure S3f), we next sought to investigate the potential correlation between the oncogene MYC and JAG2, whose expression was found unexpectedly high in Group 3 tumors. Strikingly, gene correlation analysis based on distinct MB gene expression profiles revealed a significant positive correlation of *MYC* and *JAG2* (Figure 3e and Additional file 3: Figure S3g). Furthermore, this correlation in expression was specific for Group 3 MB samples (Figure 3f and Additional file 3: Figure S3h), thus suggesting a link between one particular NOTCH ligand and the oncogene MYC in a molecularly defined patient population. Indeed, a subset of WNT MBs also possess high MYC levels [2] (Additional file 3: Figure S3f), but neither high *JAG2* expression could be detected in this subgroup (Figure 3b), nor MYC and JAG2 levels correlate among these tumors (Additional file 3: Figure S3h, middle panel). The relatively low *JAG2* expression in WNT tumors may reflect either distinct mechanisms regulating NOTCH members in this subgroup and/or a regulatory cross-talk between the WNT and NOTCH pathways in MB.

To further confirm the microarray results on *JAG2*/*MYC* expression correlation, their mRNA levels were analyzed in the same cohort of 47 primary MB samples previously examined for *JAG1* expression. Regrettably, molecular subgroup annotations were not available for this cohort of MB tumors, nevertheless *JAG2* level positively correlated with *MYC* expression (Additional file 3: Figure S3i), thus confirming the previous results and further supporting the hypothesis of the two proteins being functionally linked.

Additionally, to gain a more comprehensive overview, correlation analyses were performed also between *MYC* and the other NOTCH ligands. A slight negative correlation between *MYC* and *DLL1* was noticed (Additional file 3: Figure S3j). On the other hand, no relevant association with MYC level was detected for *JAG1*, *DLL3*, or *DLL4* in primary MB samples (data not shown), thus indicating that the association between *MYC* and *JAG2*, among the NOTCH ligands, is likely exclusive and potentially characteristic of Group 3 tumors.

### High JAG2 expression associates with aggressive anaplastic tumors and highly metastatic stages of medulloblastoma

MYC-driven MB cases (Group 3) have a high risk of recurrence, the worst outcome of the four subgroups, and a high proportion of large cells/anaplastic (LCA) tumors [27]. In comparative studies on tumor samples, as well as *in vitro* and *in vivo* preclinical investigations, the LCA variant has been associated with over-expression of the oncogene MYC and with aggressive and invasive tumor cell behavior [4]. Scoring for the association of the NOTCH ligand JAG2 with MB histological subtypes in distinct datasets with available histological details (n = 364), we detected a significant enrichment of *JAG2* expression in LCA tumors compared to classic and desmoplastic cases (Figure 3g and Additional file 4: Figure S4a). Notably, the results were similar to those obtained in the study in which the expression level of *MYC* and LCA cases were correlated (Figure 3h and Additional file 4: Figure S4b).

Moreover, because nearly 50% of Group 3 tumors are metastatic at the time of diagnosis [27], we next analyzed whether the expression of JAG2 is indicative of a higher metastatic stage in MB primary samples. In three datasets with available metastasis details (n = 172), high levels of *JAG2* were observed in highly metastatic MB samples (≥M2 stages) compared to M0 and M1 cases (Figure 3i and Additional file 4: Figure S4c). Although the limited number of highly metastatic tumors reduced the statistical relevance of these findings, similar results were obtained in the analysis in which *MYC* expression levels were examined together with M stage in the same MB samples (Figure 3j and Additional file 4: Figure S4d). Regrettably, the public datasets with available information about metastatic MB stages do not account for MB sub-grouping signatures, and vice versa. Therefore, we could not verify if high JAG2 expression correlated with MB metastatic stages in Group 3 cases specifically.

Additionally, analogous analyses examined the expression of the other NOTCH ligands in distinct histological MB subtypes and metastatic stages. However, none of the other ligands could be robustly associated with the histological features of MB tumors (data not shown). In summary, the positive and specific correlation between *MYC* and *JAG2* for LCA tumors and highly metastatic MB stages is consistent with the Group 3-specific association between *MYC* and *JAG2*. This further strengthens the notion of a functional interaction between these two proteins and suggests that high JAG2 level may be indicative of aggressive MB tumors.

### NOTCH ligand JAG2 is a MYC target gene in medulloblastoma

**Figure 4:**
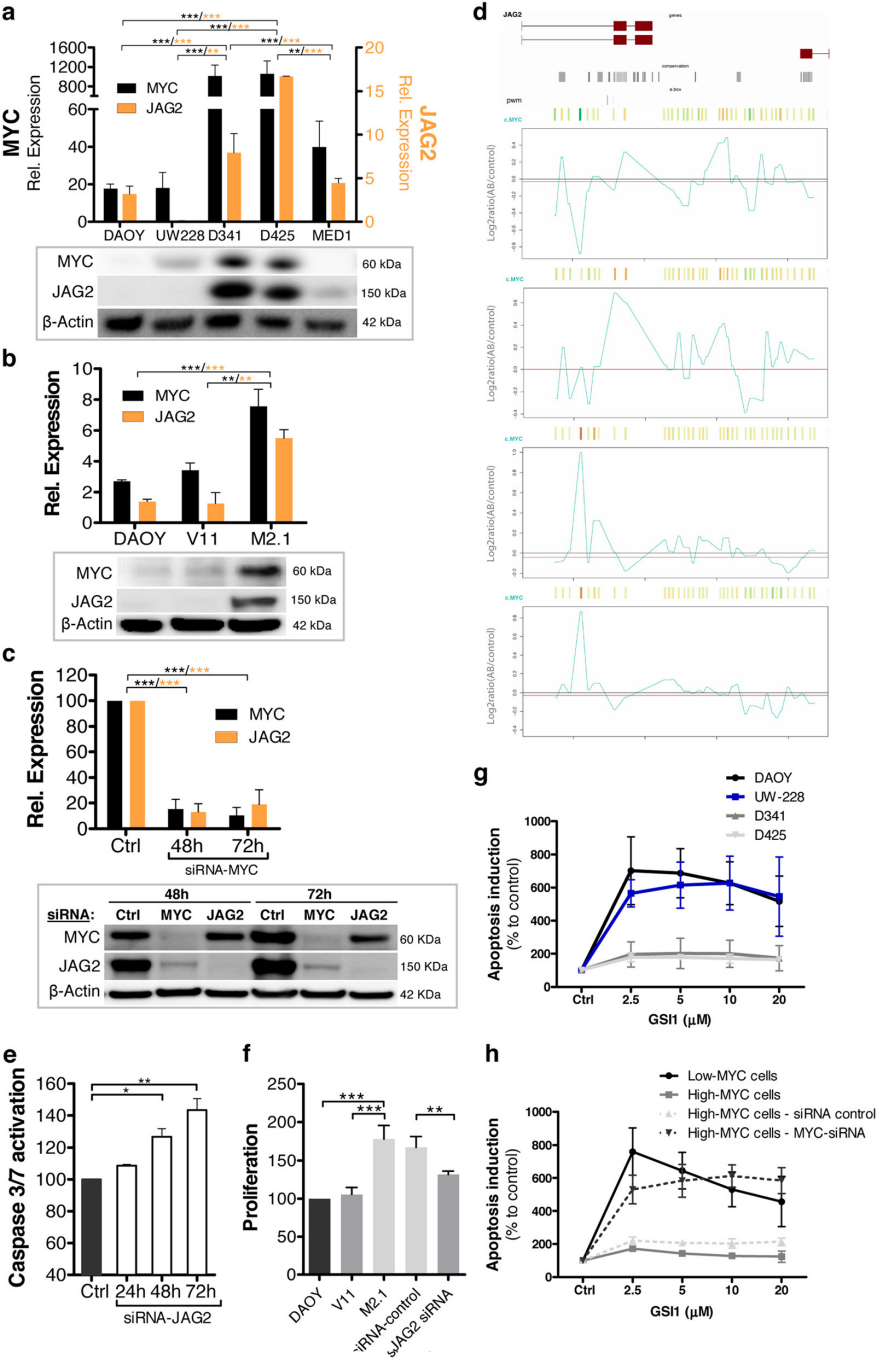
**MYC**-**dependent regulation of NOTCH ligand JAG2 expression. (a)** Relative mRNA expression (upper panel) of *MYC* (black bars, left Y axis) and *JAG2* (orange bars, right Y axis) in the indicated cell lines. Values represent the fold-change in mRNA expression relative to cerebellum (defined as 1). Protein expression (lower panel) of MYC and JAG2 in the corresponding cell lines. **(b)** Relative mRNA expression (upper panel) of *MYC* and *JAG2* in DAOY cells and stable clones of DAOY V11, and DAOY M2.1 cells. Values represent fold-change in mRNA expression relative to cerebellum (defined as 1). Protein expression (lower panel) of MYC and JAG2 in corresponding cells is shown. **(c)** MYC and JAG2 mRNA (upper panel) and protein (lower panel) expression in DAOY M2.1 cells transfected with MYC siRNA compared to control siRNA. mRNA values represent the percent decrease in *JAG2* and *MYC* expression relative to control. Expression of β-actin was used as a control for western blot analysis. Statistical analysis: black stars indicate p values relative to MYC expression; orange stars indicate p values relative to JAG2 expression. **(d)** ChIP-on-chip data show occupancy of the *JAG2* genomic sequence by MYC in four MB cell lines (from top to bottom: DAOY, UW-228, D458, and D341). **(e)** Time-dependent caspase 3/7 activation in DAOY M2.1 cells upon JAG2 siRNA treatment at the indicated time-points; values represent the percent increase in caspase 3/7 activity relative to control. **(f)** Proliferation status of DAOY cells, DAOY V11, DAOY M2.1, and DAOY M2.1 cells at 48 hours after JAG2 siRNA treatment compared to control siRNA. Caspase 3/7 activation at 48 hours after treatment with GSI in the indicated cell lines **(g)** and DAOY-derived clones **(h)**. Low MYC cells: DAOY; high MYC cells: DAOY M2.1. Values represent the percent increase in caspase 3/7 activity relative to control siRNA.

Given the extensive evidence correlating *MYC* and *JAG2*, we next investigated in greater detail the mechanistic link between this NOTCH ligand and the oncogene. To assess whether in MB JAG2 expression is MYC-dependent, first we compared JAG2 and MYC levels in a panel of human MB cell lines. Remarkably, JAG2 expression was high at the mRNA and protein levels in the high-expressing MYC cell lines (D341 and D425), whereas JAG2 levels were lower in the low-expressing MYC MB cell lines (DAOY and UW-228) (Figure 4a). MYC-dependent JAG2 expression was experimentally further confirmed in MB cells genetically manipulated to have either increased or decreased MYC expression. In MB cells stably transfected with a MYC expression construct (DAOY M2.1), both the mRNA and protein levels of JAG2 were considerably higher compared to wild-type- and empty vector-transfected cells (Figure 4b). Increased JAG2 expression in M2.1 cells was indeed MYC-dependent because MYC overexpression-induced JAG2 levels were blunted following MYC depletion by siRNA (Figure 4c). Unexpectedly, after 72 hours of JAG2 silencing a moderate decrease in MYC protein abundance was observed. However, the analysis of MYC mRNA level following JAG2 siRNA showed a very small and statistically in-significant decrease in expression only after 72 hours (Additional file 4: Figure S4e). Because alteration of MYC expression did not take place at earlier time-points, these results suggested that the observed effect at the protein level is likely independent of transcriptional regulation.

Furthermore, to verify if the oncogene MYC can modulate the transcription of NOTCH ligand JAG2, we examined by chromatin immunoprecipitation whether MYC, as transcription factor, was bound to the promoter region of *JAG2* in a panel of MB cell lines. Genomic DNA was extracted and precipitated with a MYC-specific antibody to enrich MYC-binding promoter sequences, which were then hybridized to a promoter oligo-array [29]. An enrichment of DNA fragments surrounding the transcriptional start site of *JAG2* was detected (Figure 4d and Additional file 5: Figure S5a), indicating that MYC is indeed able to bind the *JAG2* promoter region and thus potentially capable of modulating its expression. Notably, higher amounts of MYC protein were detected at the *JAG2* promoter in a subset of cell lines with high MYC levels (*e.g*., D341 and D458 cells) compared to those with low MYC levels (*e.g*., DAOY and UW-228). In agreement with recent findings describing MYC as a universal amplifier rather than an on-off transcriptional switcher [32], our experimental evidence suggests that in tumor cells expressing high levels of MYC, this transcription factor accumulates in the promoter regions of (already) active genes, likely further increasing the levels of transcripts within the cell’s gene expression program. Thus, the identification of *JAG2* as MYC target gene suggests that constitutive MYC induction could be involved in the alteration of NOTCH-related developmental programs in MB.

### The oncogene MYC alters NOTCH signaling in medulloblastoma

Finally to evaluate if JAG2 has a functional role in MYC-driven MB pathogenesis, we examined the biological function of JAG2 in MB cells expressing high levels of MYC (DAOY M2.1) [20]. Upon JAG2 depletion by siRNA transfection in cells with high MYC expression (Additional file 5: Figure S5b), the number of cells undergoing apoptosis was slightly increased (Figure 4e), while proliferation concomitantly decreased (Figure 4f). Since MYC induction in MB cells leads to an increased proportion of cells undergoing programmed cell death, while overall viability is maintained through higher rate of proliferation [20], our results suggested that JAG2 might be one of the proteins involved in the regulation of MYC-controlled apoptosis or proliferation. However, the mechanism underlying this regulation remains incompletely understood. In fact, the levels of NICD2 and of NOTCH target HES1 were not altered upon JAG2 silencing (Additional file 5: Figure S5c), indicating that JAG2 functions are independent of canonical NOTCH cascade activation. Moreover, although JAG2 depletion in MB cells expressing high levels of MYC led to apoptosis and reduced proliferation, unexpectedly, these phenotypical alterations were not mirrored by reduced cell viability (Additional file 5: Figure S5d). Besides, we noticed that JAG2 depletion caused a significant increase in the relative mRNA and protein levels of JAG1 (Additional file 5: Figure S5e). Analogous effects were observed when JAG2 levels were measured under conditions in which JAG1 was depleted (data not shown). Consistently, MB cell lines with high expression of JAG2/MYC (D341 and D425; Figure 4a) also displayed lower JAG1 levels compared to cells expressing low MYC levels (DAOY and UW-228; Figure 2b). Thus, it is possible that these two ligands are mutually regulated in a manner in which the high abundance of one ligand represses the expression of the other ligand and vice-versa. Hence, increased JAG1 expression induced by JAG2 depletion could provide protection from apoptosis and thereby increase the viability of JAG2 knocked-down cells. The inverse correlation between JAG1 and JAG2 expression was additionally verified by the analysis of MB cells bearing different level of MYC/JAG2. In high-MYC cells (DAOY M2.1), parallel with the high *JAG2* expression induced by MYC, *JAG1* expression was lower (Additional file 5: Figure S5f). Consistently, *JAG1* level increased upon JAG2 reduction triggered by MYC knockdown (Additional file 5: Figure S5g). Interestingly, in cells expressing high levels of MYC/JAG2, the expression of the NOTCH target *HES1* was considerably decreased (Additional file 5: Figure S5f), whereas *HES1* expression was up-regulated when MYC was absent and *JAG1* was expressed (Additional file 5: Figure S5g). These findings highlighted the key role of JAG1 in canonical NOTCH activation, and further suggested that JAG2 signals through a non-canonical NOTCH pathway.

Finally, because MYC controls JAG2 expression and thereby likely alters NOTCH signaling, we sought to determine whether the expression levels of MYC in MB cells impact the response to treatment with γ-secretase inhibitor (GSI), a small molecule able to block the NOTCH cascade. Indeed, GSI treatment at concentrations which proved to be effective in inhibiting NOTCH cascade in MB cells [33], showed varying responses in different MB cell lines, correlating with the level of MYC expression. MB cells overexpressing MYC were less sensitive to treatment-induced apoptosis compared to low MYC cells (Figure 4g and h) and, strikingly, sensitivity to GSI-induced apoptosis was restored when MYC was depleted (Figure 4h). Altogether these results indicated the existence of a finely tuned regulatory mechanism that results in mutual regulation of JAG1 and JAG2 expression in MB cells, and suggested that the oncogene MYC is able to influence the NOTCH cascade, at least partially via the transcriptional induction of JAG2 expression.

## Discussion

This study shed light on the yet unexplored pathological functions of NOTCH ligands in MB, and verified our main hypothesis that an alteration of NOTCH developmental pathway may be caused by abnormal ligand expression. By examining the expression levels in large cohorts of MB primary tumors and established *in vitro* MB models, we demonstrated the presence of abnormal levels of four distinct NOTCH ligands (JAG1, JAG2, DLL1, and DLL4) in MB. By analyzing their patterns of expression across MB molecular subgroups, we showed that NOTCH ligand JAG1 is broadly over-expressed in MBs, and homogeneously distributed across subgroups. In contrast, JAG2 is generally under-expressed in tumors compared to normal cerebellum, but a subpopulation of MYC-driven MBs bear increased levels of this ligand. Importantly, MYC/JAG2 correlation is specific for Group 3 cases, therefore suggesting a link between one particular NOTCH ligand and the oncogene MYC in a molecularly defined patient population. By identifying JAG2 as MYC target, and by showing that MB cells acquire induced expression of JAG2 through MYC-induced transcriptional activation, we described for the first time a mechanistic link between the oncogene MYC and NOTCH pathway in MB. These results are supported by the report from Yustein et al. that described JAG2 as one of the MYC target genes participating in tumorigenesis in a human B cell model [34]. Together with a study describing NOTCH4 as a MYC target [35], these two reports represent the only experimental evidence, to our knowledge, for transcriptional control of the NOTCH pathway by MYC.

Moreover, the positive and specific correlation of *MYC* and *JAG2* with aggressive anaplastic tumors, and highly metastatic MB stages might represent a clinically relevant finding. Because future stratification of MB patients will likely involve the inclusion of phenotypic tumor cell parameters, these results suggest that determining the expression level of JAG2 may be helpful for the sub-classification of MYC-driven MB to distinguish aggressive tumors from less severe malignances. From the clinical point of view, these results may also be relevant for current histology-based diagnosis of MB. Indeed, the identification of LCA MB will likely retain its prognostic significance, even when molecular sub-grouping will more frequently be used in clinics; in this context, JAG2 may represent an additional potential marker for high MYC/LCA MB tumors. Regrettably, the number of MB samples collected in the Children’s Hospital of Zurich is too small to conduct a meaningful survival analysis. Moreover, the public datasets of primary MB samples, from where the data for this study was extracted, lack survival data. Therefore, we could not perform a survival analysis comparing patients with or without aberrant JAG1 or JAG2.

In addition, current MB research is intensely focusing on developing accurate mouse models of MYC-driven MB. To overcome MYC-induced apoptosis, two recently developed MYC-driven mouse MB models require loss of p53 [36,37]. Because JAG2 appeared to cooperate with MYC to protect MYC-overexpressing cells from apoptosis, JAG2 induction/up-regulation could be a useful strategy for the development of such high-MYC-expressing MB animal models.

**Figure 5:**
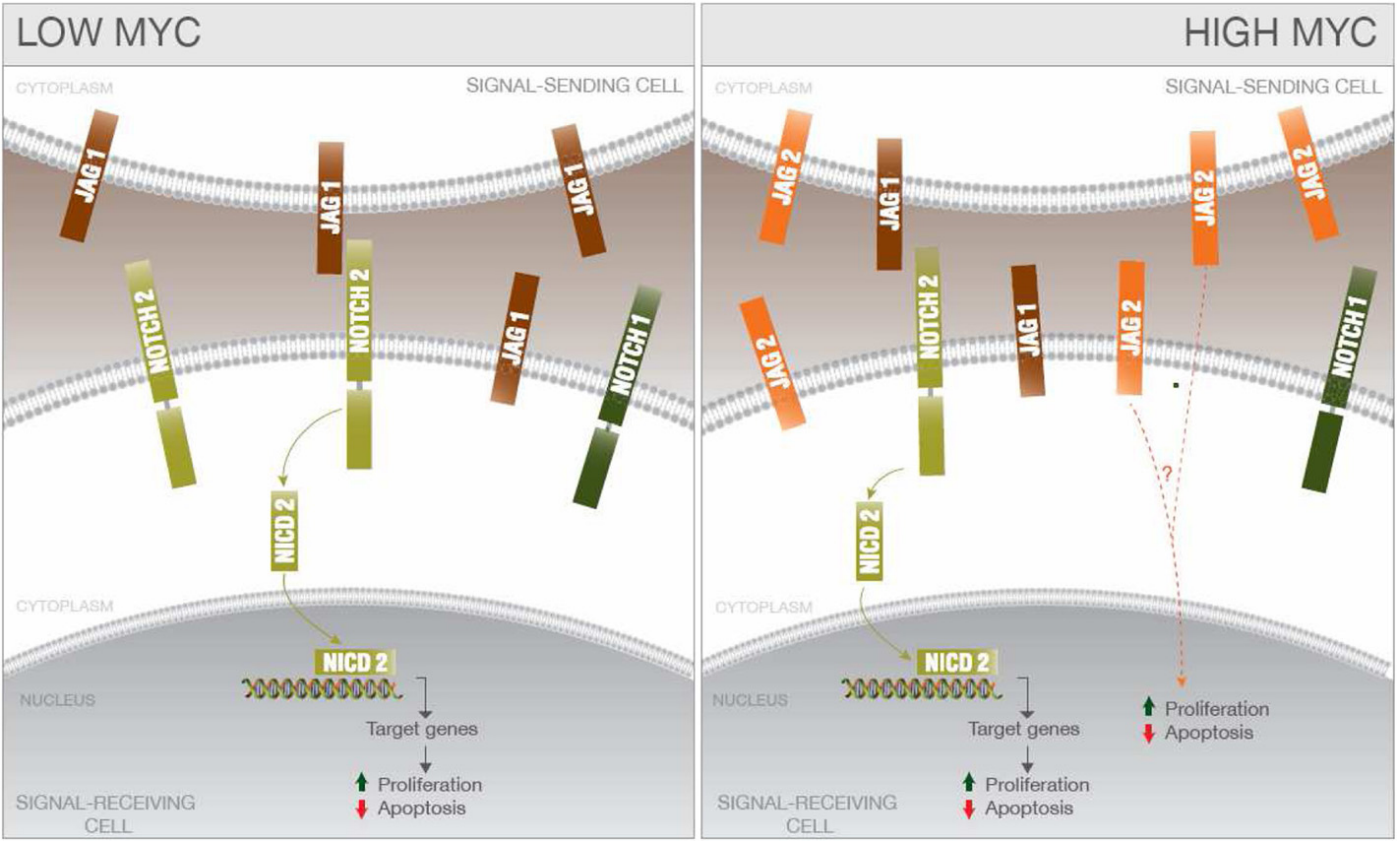
**MYC**-**dependent NOTCH signaling in MB cells.** Simplified scheme illustrating the MYC-dependent NOTCH molecular network involving JAG1 and JAG2 in MB cells. (Left panel) Low MYC MB cell. (Right panel) High MYC MB cell.

To summarize, we propose a simplified model that illustrates NOTCH signaling induced by JAG1 and JAG2 in MB cells in the context of different levels of MYC (Figure 5). The majority of MB tumors bear low MYC levels and high levels of JAG1, which triggers pro-proliferative signaling through NOTCH2. On the other hand, a subset of MBs, and/or a subset of cells within a given tumor, express high levels of MYC and acquire a concomitantly increased JAG2 protein level, which likely alters the fine-tuned NOTCH signaling cascade.

In line with previous reports showing in different tumor models that the inhibition of NOTCH ligands has proven effective [15,38], our study showed that interfering with activation of the NOTCH pathway by targeting its ligands may represent a new direction for alternative therapeutic approaches against MB. In particular, targeting JAG1 appears to be a promising strategy. JAG1 is highly expressed in the vast majority of MBs and homogeneously distributed across subgroups; moreover, JAG1 depletion inhibited pro-proliferative NOTCH2 signals and caused a decrease in expression of HES1, which plays a central role in MB pathogenesis [39-42]. Furthermore, NOTCH1-mediated signaling has been associated with activation of an anti-proliferative cascade in MB [17]; therefore, by specifically inhibiting the JAG1-NOTCH2 axis, a survival signaling would be specifically blocked, and side effects due to broad and non-specific NOTCH inhibition could be avoided. Indeed, such treatment would specifically target a cell membrane protein that is overexpressed in MB tumors, but not in normal cerebellum, thus likely reducing treatment-related side effects on the developing brains of children.

Recent approaches attempting to block NOTCH signaling via inhibitors of the γ-secretase have been effective and these compounds are currently being tested in clinical trials for the treatment of brain tumors, including MBs [43]. However, γ-secretase inhibitors developed thus far broadly inhibit γ-secretases and are unable to distinguish individual NOTCH ligand/receptor interactions; as a result, they also affect other pathways [44], and such treatment is associated with intestinal toxicity [45]. Besides, in MB a broad NOTCH inhibition will likely also block anti-proliferative signals induced by the NOTCH1 receptor [17]. These negative, treatment-related consequences could be minimized by therapeutic strategies specifically targeting individual ligands or receptors. In addition, we observed that the response of MB cells to GSI treatment was influenced by MYC status, although the mechanism remains incompletely understood. Because MYC depletion reduces JAG2 and increases JAG1 expression, thereby shifting cells from non-canonical to canonical NOTCH signaling, it is conceivable that the dependence on canonical NOTCH signaling would be increased. This observation is significant because JAG2 blockage in MYC-driven MB tumors may cause the re-acquisition of tumor sensitivity to treatment with γ-secretase inhibitors.

Therefore, further studies are required to determine the benefits of such treatments in combination with NOTCH ligand inhibition for different MB subtypes, particularly MYC-driven tumors. Additional effort is also needed for the development of neutralizing antibodies and/or small molecules targeting specific NOTCH ligands, which should then be tested in MB animal models to verify the benefits of such treatments.

## Conclusion

This study advanced our understanding of NOTCH signaling and its pathological role in MB, and investigated for the first time the therapeutic benefits of interfering with NOTCH pathway via the inhibition of NOTCH ligands in MB cells. This approach represents an attractive strategy to be considered in combination with targeting SHH or WNT pathways or as a side therapy to synergize with the apoptosis-inducing effects of standard chemo-therapeutics.

## Competing interests

The authors declare that they have no competing interests.

## Authors’ contributions

GF, TS, MB, and MAG, conceived and designed the experiments. GF completed and analyzed all experiments except those specifically listed here. CS and FW provided and analyzed the ChIP-on-chip data. DC and AA assisted with the manuscript. GF, TS, MB, and MAG wrote the manuscript. All authors read and approved the final manuscript.

## Supplementary Material

Additional file 1: Figure S1 Expression of NOTCH ligands in MB primary tumors and cerebellum samples. **(a)** Relative mRNA expression of the indicated NOTCH ligands in two independent gene expression datasets of human MB tumors [Left panel: 57 samples [22]; right panel: 76 samples [26]. **(b)** Relative mRNA expression of the indicated NOTCH ligands in a gene expression profile dataset of human cerebellum samples (n = 9) [28]. Click here for file

Additional file 2: Figure S2 JAG1 siRNA-mediated silencing and correlation of *JAG1*/*HES1* in primary MB tumors. **(a)** Relative *JAG1* mRNA expression in DAOY cells upon JAG1 siRNA treatment at the indicated time-points. Values represent the percent decrease in *JAG1* mRNA relative to the control. **(b)** Correlation between *JAG1* and *HES1* mRNA expression in three representative datasets of human MB tumors: left panel, 76 samples [26]; middle panel, 57 samples [22]; right panel, 62 samples [23]. r: pearson’s value; p: p values. **(c)** Western blot showing expression of JAG1 and NICD1 in DAOY cells at 48 hours after JAG1 siRNA treatment compared to control siRNA. β-actin expression was used as control. Click here for file

Additional file 3: Figure S3 Validation of the expression of NOTCH ligands across MB molecular subgroups and correlation of *MYC*/*JAG2* expression in MB primary tumors. Dot plots showing the relative expression (log 2) of *JAG1***(a)**, *JAG2***(b)**, *DLL1***(c)**, *DLL3***(d)**, and *DLL4***(e)** across MB subgroups in 285 human MB tumors [24]: SHH, n = 51; Grp3 (Group 3), N = 46; Grp4 (Group 4), n = 188. **(e** and **f)** Dot plots showing the relative expression of *DLL4* and *MYC*, respectively, across MB subgroups in two datasets. Left panel [25]: SHH, n = 33; WNT, n = 8; Grp3 (Group 3), n = 27; Grp4 (Group4), n = 35. Right panel [24]: SHH, n = 51; Grp3 (Group 3), n = 46; Grp4 (Group 4), n = 188. **(g)** Correlation between *MYC* and *JAG2* mRNA expression in 285 MB tumors [24]. r: Pearson’s value; p: p values. **(h)** Correlation study of *JAG2* and *MYC* expression levels (log 2) across MB subgroups (SHH, WNT, Group 4) [25]. **(i)** Correlation between *MYC* and *JAG2* mRNA expression (log 2) in 47 MB primary samples. **(j)** Correlation between *MYC* and *DLL1* mRNA expression in two datasets of 285 MB tumors (left panel) [24] and 103 MB tumors (right panel) [25]. r: Pearson’s value; p: p values. Click here for file

Additional file 4: Figure S4 High JAG2 expression in LCA MB tumors and highly metastatic MB cases. Box plots showing *JAG2***(a)** and *MYC***(b)** expression (log 2) according to MB histological variants of MB tumors. Left panels (n = 251) [24]: classic (n = 200), desmoplastic (desmop.) (n = 21), and (LCA) large cells/anaplastic (n = 30). Right panels (n = 103) [25]: classic (n = 77), desmoplastic (desmop.) (n = 16), and (LCA) large cells/anaplastic (n = 8); center line = median. Box plots showing *JAG2***(c)** and *MYC***(d)** expression in MB tumors clustered by the metastatic stage of MB tumors; center line = median. Left panels (n = 63): M0, n = 45; M1, n = 5; M2, n = 4; M3, n = 9. Right panels (n = 46) [21]: M0, n = 26; M1, n = 7; ≥M2, n = 13. **(e)** MYC mRNA expression in DAOY M2.1 cells upon JAG2 siRNA at the indicated time-points. mRNA values represent the percent decrease in *MYC* expression relative to siRNA control. Click here for file

Additional file 5: Figure S5 Validation of MYC-dependent JAG2 expression and compensatory mechanism regulating relative JAG1/JAG2 levels. **(a)** MYC binding to the JAG2 promoter in four additional MB cell lines. ChIP-on-chip data showing occupancy of the *JAG2* genomic sequence by MYC in four additional MB cell lines (from top to bottom: D283, ONS76, PNET5, and MED8A). **(b)** Relative *JAG2* mRNA expression in MYC stably transfected DAOY M2.1 cells upon JAG2 siRNA treatment at the indicated time-points. **(c)** Western blot showing the expression of JAG2, NICD2, and HES1 in MYC stably transfected DAOY M2.1 cells at 48 hours after JAG2 siRNA treatment compared to control siRNA; β-actin expression was used as a control. **(d)** Cell viability of MYC stably transfected cells (DAOY M2.1) at 48 hours after JAG2 siRNA treatment compared to control siRNA. **(e)** Relative JAG1 and JAG2 mRNA expression (left panel) and protein expression (right panel) in DAOY M2.1 cells at 72 hours after JAG2 siRNA treatment. **(f)** Relative *JAG1* and *HES1* mRNA expression in DAOY M2.1 MYC stably transfected cells (high MYC) and DAOY V11 empty vector-transfected cells (low MYC). **(g)** Relative *JAG1* and *HES1* mRNA expression in DAOY M2.1 cells at 48 hours after MYC siRNA compared to control siRNA. Click here for file
