# Supplementary material for: NOTCH ligands JAG1 and JAG2 as critical pro-survival factors in childhood medulloblastoma
Source: Acta Neuropathol Commun. 2014 Apr 7;2:39. doi: 10.1186/2051-5960-2-39 (PMC4023630; doi:10.1186/2051-5960-2-39)

Article Title:
NOTCH ligands JAG1 and JAG2 as critical pro-survival factors in childhood medulloblastoma

Journal: *Acta Neuropathologica Communications*

Authors:
Giulio Fiaschetti, Christina Schroeder, Deborah Castelletti, Alexandre Arcaro, Frank Westermann, Martin Baumgartner, Tarek Shalaby, Michael A. Grotzer*

*Corresponding author

Prof. Dr. Michael A. Grotzer
Department of Oncology, University Children's Hospital of Zurich, Switzerland
Email: Michael.Grotzer@kispi.uzh.ch

Supplementary Figure S1

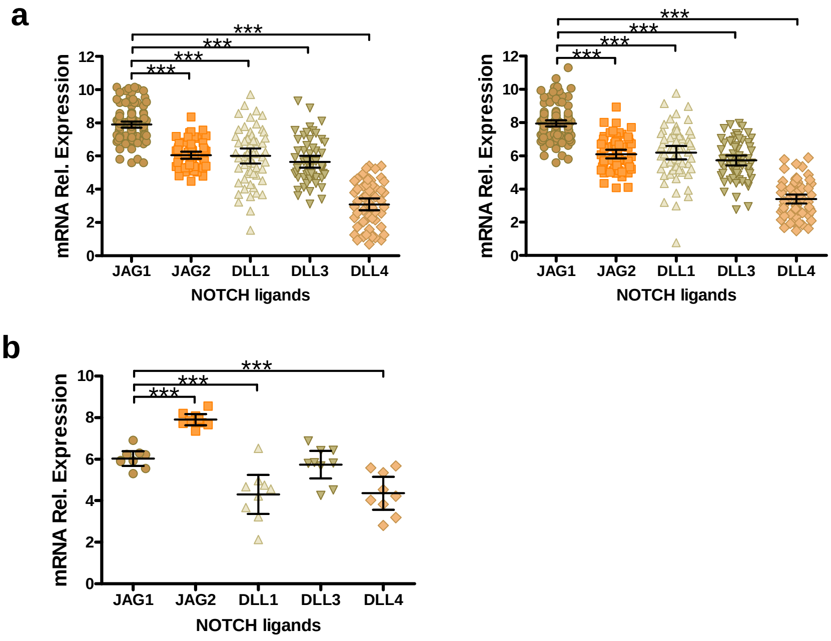

Supplement: Additional file 1: Figure S1 — Expression of NOTCH ligands in MB primary tumors and cerebellum samples. (a) Relative mRNA expression of the indicated NOTCH ligands in two independent gene expression datasets of human MB tumors [Left panel: 57 samples [22]; right panel: 76 samples [26]. (b) Relative mRNA expression of the indicated NOTCH ligands in a gene expression profile dataset of human cerebellum samples (n = 9) [28]. [file 2051-5960-2-39-S1.pdf]
