# Supplementary figures and images for: NOTCH ligands JAG1 and JAG2 as critical pro-survival factors in childhood medulloblastoma
Source: Acta Neuropathol Commun. 2014 Apr 7;2:39. doi: 10.1186/2051-5960-2-39 (PMC4023630; doi:10.1186/2051-5960-2-39)

Supplementary Figure S2

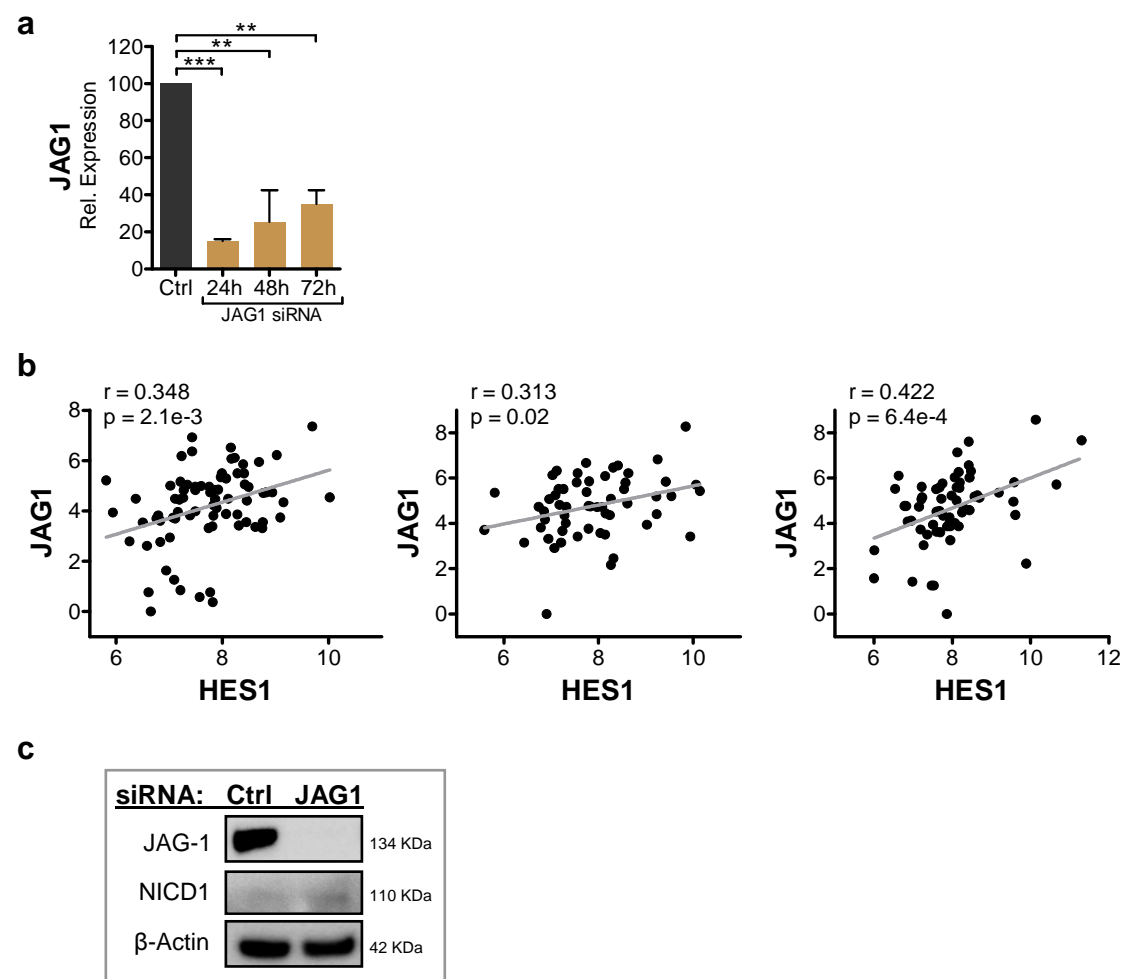

Supplement: Additional file 2: Figure S2 — JAG1 siRNA-mediated silencing and correlation of JAG1/HES1 in primary MB tumors. (a) Relative JAG1 mRNA expression in DAOY cells upon JAG1 siRNA treatment at the indicated time-points. Values represent the percent decrease in JAG1 mRNA relative to the control. (b) Correlation between JAG1 and HES1 mRNA expression in three representative datasets of human MB tumors: left panel, 76 samples [26]; middle panel, 57 samples [22]; right panel, 62 samples [23]. r: pearson’s value; p: p values. (c) Western blot showing expression of JAG1 and NICD1 in DAOY cells at 48 hours after JAG1 siRNA treatment compared to control siRNA. β-actin expression was used as control. [file 2051-5960-2-39-S2.pdf]

Supplementary Figure S3

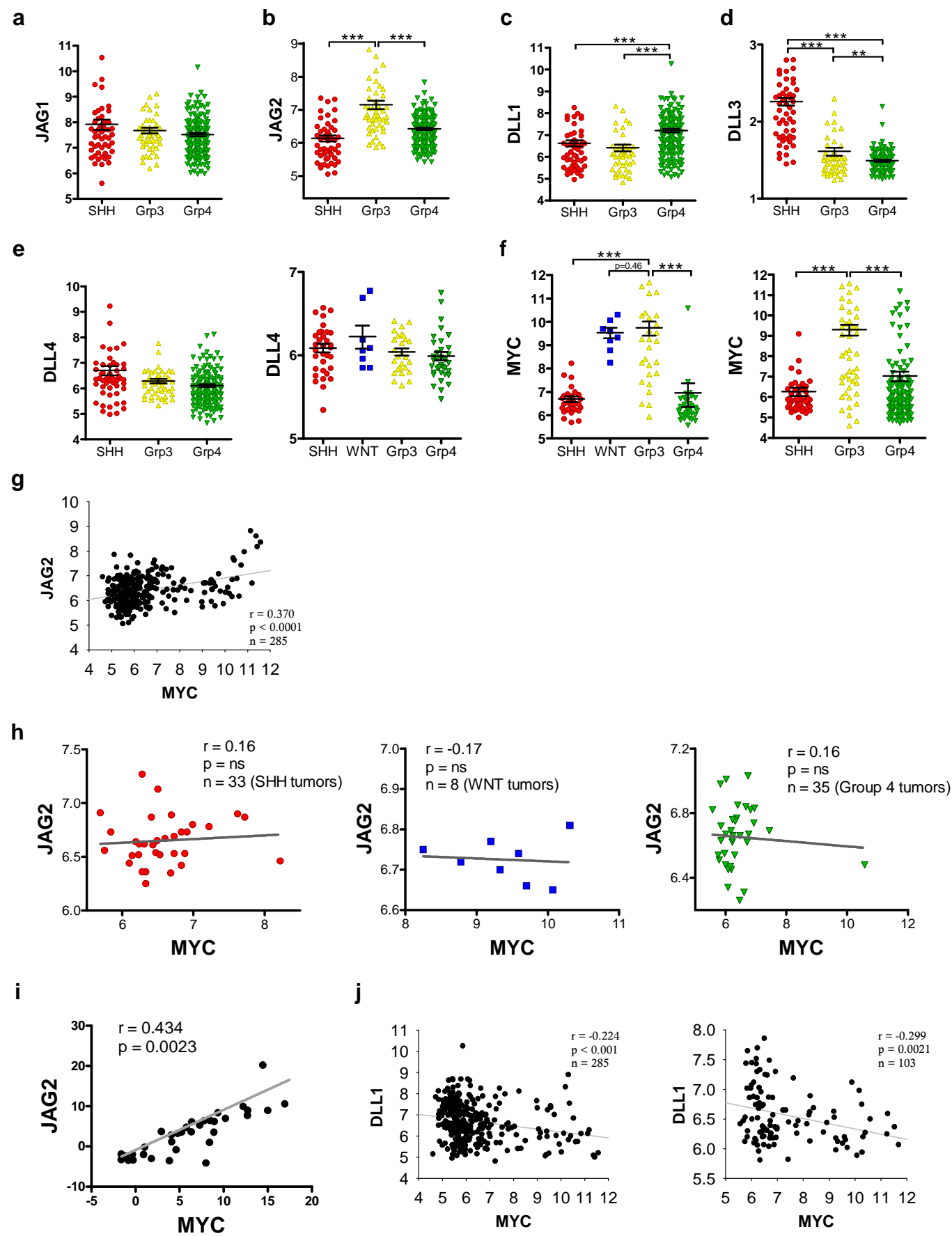

Supplement: Additional file 3: Figure S3 — Validation of the expression of NOTCH ligands across MB molecular subgroups and correlation of MYC/JAG2 expression in MB primary tumors. Dot plots showing the relative expression (log 2) of JAG1(a), JAG2(b), DLL1(c), DLL3(d), and DLL4(e) across MB subgroups in 285 human MB tumors [24]: SHH, n = 51; Grp3 (Group 3), N = 46; Grp4 (Group 4), n = 188. (e and f) Dot plots showing the relative expression of DLL4 and MYC, respectively, across MB subgroups in two datasets. Left panel [25]: SHH, n = 33; WNT, n = 8; Grp3 (Group 3), n = 27; Grp4 (Group4), n = 35. Right panel [24]: SHH, n = 51; Grp3 (Group 3), n = 46; Grp4 (Group 4), n = 188. (g) Correlation between MYC and JAG2 mRNA expression in 285 MB tumors [24]. r: Pearson’s value; p: p values. (h) Correlation study of JAG2 and MYC expression levels (log 2) across MB subgroups (SHH, WNT, Group 4) [25]. (i) Correlation between MYC and JAG2 mRNA expression (log 2) in 47 MB primary samples. (j) Correlation between MYC and DLL1 mRNA expression in two datasets of 285 MB tumors (left panel) [24] and 103 MB tumors (right panel) [25]. r: Pearson’s value; p: p values. [file 2051-5960-2-39-S3.pdf]

Supplementary Figure S4

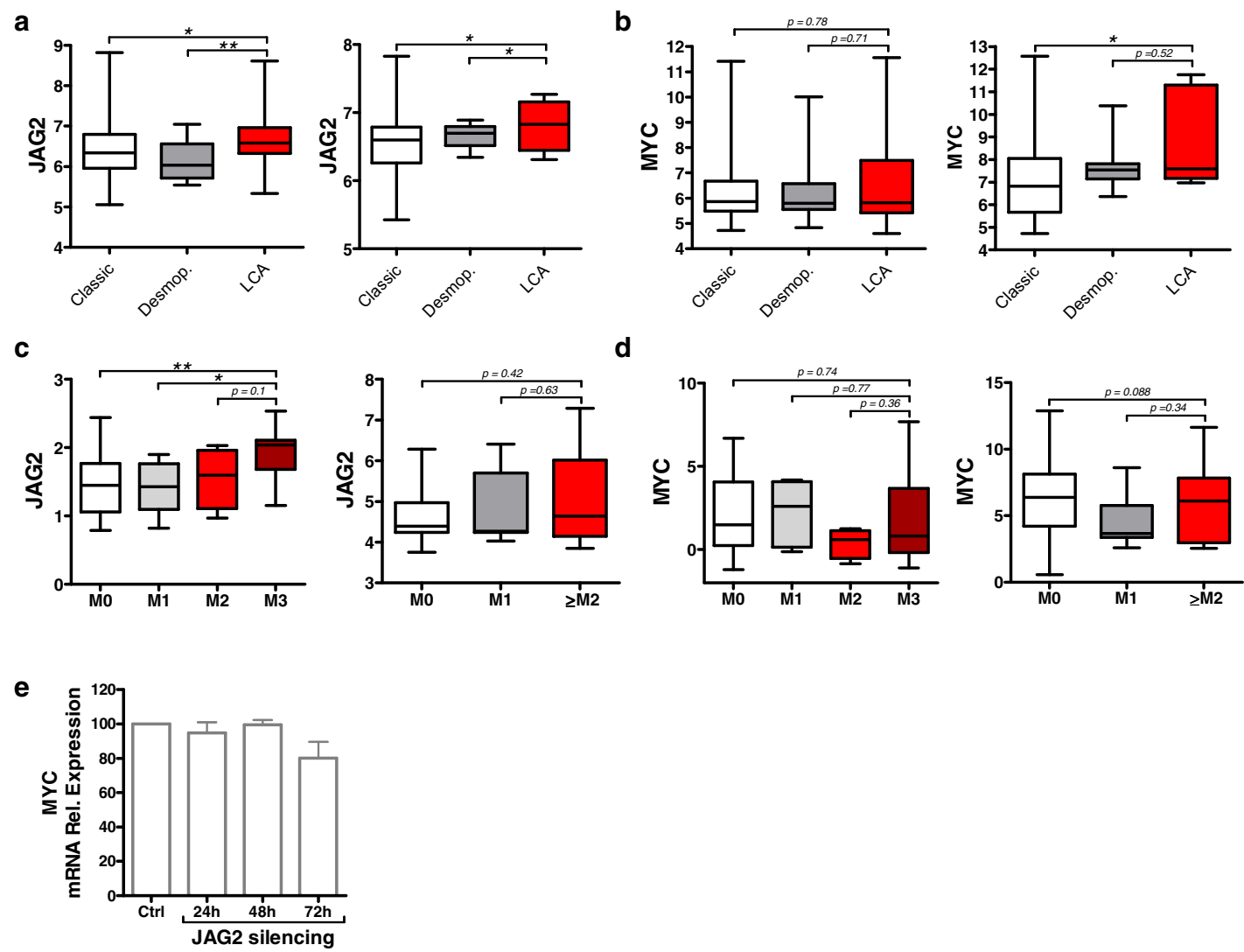

Supplement: Additional file 4: Figure S4 — High JAG2 expression in LCA MB tumors and highly metastatic MB cases. Box plots showing JAG2(a) and MYC(b) expression (log 2) according to MB histological variants of MB tumors. Left panels (n = 251) [24]: classic (n = 200), desmoplastic (desmop.) (n = 21), and (LCA) large cells/anaplastic (n = 30). Right panels (n = 103) [25]: classic (n = 77), desmoplastic (desmop.) (n = 16), and (LCA) large cells/anaplastic (n = 8); center line = median. Box plots showing JAG2(c) and MYC(d) expression in MB tumors clustered by the metastatic stage of MB tumors; center line = median. Left panels (n = 63): M0, n = 45; M1, n = 5; M2, n = 4; M3, n = 9. Right panels (n = 46) [21]: M0, n = 26; M1, n = 7; ≥M2, n = 13. (e) MYC mRNA expression in DAOY M2.1 cells upon JAG2 siRNA at the indicated time-points. mRNA values represent the percent decrease in MYC expression relative to siRNA control. [file 2051-5960-2-39-S4.pdf]

Supplementary Figure S5

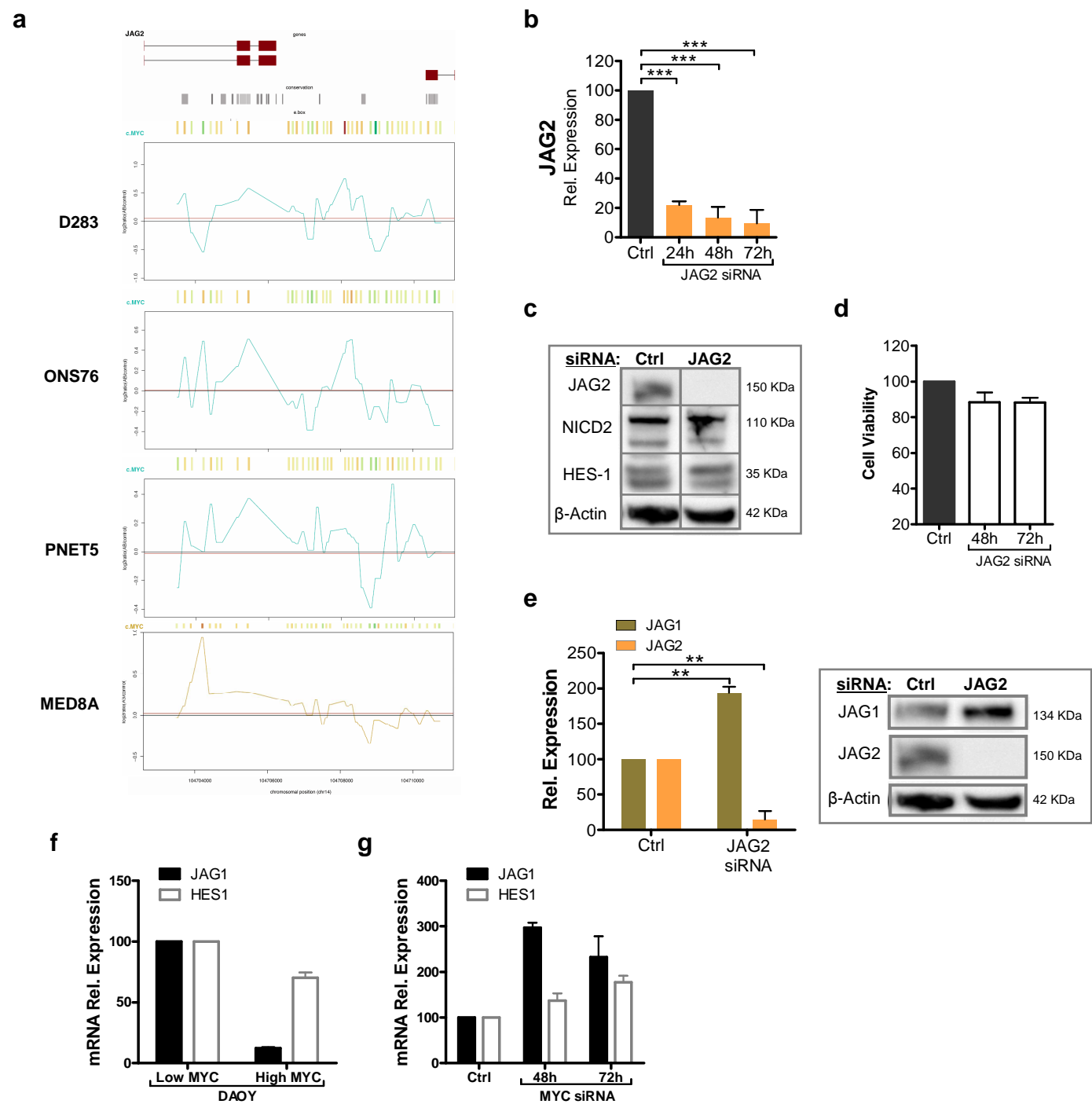

Supplement: Additional file 5: Figure S5 — Validation of MYC-dependent JAG2 expression and compensatory mechanism regulating relative JAG1/JAG2 levels. (a) MYC binding to the JAG2 promoter in four additional MB cell lines. ChIP-on-chip data showing occupancy of the JAG2 genomic sequence by MYC in four additional MB cell lines (from top to bottom: D283, ONS76, PNET5, and MED8A). (b) Relative JAG2 mRNA expression in MYC stably transfected DAOY M2.1 cells upon JAG2 siRNA treatment at the indicated time-points. (c) Western blot showing the expression of JAG2, NICD2, and HES1 in MYC stably transfected DAOY M2.1 cells at 48 hours after JAG2 siRNA treatment compared to control siRNA; β-actin expression was used as a control. (d) Cell viability of MYC stably transfected cells (DAOY M2.1) at 48 hours after JAG2 siRNA treatment compared to control siRNA. (e) Relative JAG1 and JAG2 mRNA expression (left panel) and protein expression (right panel) in DAOY M2.1 cells at 72 hours after JAG2 siRNA treatment. (f) Relative JAG1 and HES1 mRNA expression in DAOY M2.1 MYC stably transfected cells (high MYC) and DAOY V11 empty vector-transfected cells (low MYC). (g) Relative JAG1 and HES1 mRNA expression in DAOY M2.1 cells at 48 hours after MYC siRNA compared to control siRNA. [file 2051-5960-2-39-S5.pdf]
